# Electrolyte disturbances in a sample of hospitalized patients from Iraq

**DOI:** 10.25122/jml-2022-0039

**Published:** 2022-09

**Authors:** Suad Lateef Ibrahim, Zubaida Falih Alzubaidi, Fatma Abbas Dawood Al-Maamory

**Affiliations:** 1 Department of Clinical Laboratory Sciences, Faculty of Pharmacy, University of Kufa, Kufa, Iraq; 2 Laboratories of Al-Imamain Al-Kadhimain Medical City, Ministry of Health, Baghdad, Iraq

**Keywords:** electrolyte disturbances, ICU, RCU, CCU

## Abstract

Electrolyte disturbances are common in ill patients. Several conditions in the intensive care unit (ICU) might be responsible for developing electrolyte disorders, and medications may also contribute to these disturbances. The current study aimed to determine the frequency of electrolyte disturbances and assess the pattern of electrolyte imbalance in hospitalized patients, determining the possible effects of these electrolyte disorders. This cross-sectional study included patients admitted to the intensive care unit, respiratory care unit (RCU), and coronary care unit (CCU) at the Al-Sadar teaching hospital, Najaf, Iraq, from November 2020 to April 2021. The study collected data from two hundred patients regarding demographics, categories of ICUs at admission, comorbidities, and laboratory values at admission. Also, electrolyte levels at ICU admission and during hospitalization were collected from the medical database record. In addition, the patient's age, sex, fasting blood sugar (FBS), body mass index (BMI), B.urea, and creatinine were matched. Na^+^, K^+^, ionized Ca^++^, and Cl serum levels were significantly different during hospitalization. Comorbidities with predominant hypokalemia were found in 80.5%, hypochloremia in 73%, hypocalcaemia in 72%, and hyponatremia in 56.7% of hospitalized patients. Studying the effect of co-morbidities indicated a higher percentage (44%) of admitted patients with ischemic heart diseases, 38 (19%) with digestive diseases, 21 (10.5%) with orthopedic surgery in an emergency, 14 (7%) with pneumonia and lung diseases, 12 (6%) with diabetics, 18 (9%) with sepsis, and 9(4.5%) with seizure. Hospitalized patients may be at higher risk of developing combined electrolytes disorder associated with decreased serum levels of K^+^, Na^+^, Ca^++^, and Cl^-^. Thus, doctors and clinicians are recommended to observe electrolyte changes and correct them as they seem to negatively impact the outcome and prognosis.

## INTRODUCTION

Electrolytes are involved in many homeostatic and metabolic functions that include biochemical and enzymatic reactions to maintain hormone function, cardiovascular function, cell membrane and structure-function, nerve signal conduction, neurotransmission, muscle contraction, fluid and acid-base regulation, and bone composition. Abnormalities of electrolytes associated with the appearance of signs and symptoms should be monitored in patients with electrolyte disturbances. These symptoms are related to disorder severity and rate of disorder development [1]. The disorder of electrolytes can be considered one of the most pressing medical issues in the ICU and other hospitalized patients. It has been reported that electrolyte imbalances are associated with increased morbidity and mortality among ill patients [2].

Electrolyte disorders may occur due to many diseases, including diabetes mellitus, acute renal failure, chronic renal failure or rheumatoid factor, cardiovascular disease, and shock [3–5]. However, electrolytes are usually determined routinely from laboratory tests [6]. Fluids and electrolyte disturbances were associated with various conditions such as heart failure, brain damage, sepsis, trauma, and severe burns. Many mechanisms are involved in inducing electrolyte abnormalities in ICU, including alteration of electrolyte absorption and distribution, changes in fluid status and shits, hormonal alterations, inadequate or excessive fluid administration, and neurologic alteration of excretion via gastrointestinal (GI) or renal losses [1].

Intensive care unit (ICU) services require expensive technology and account for 10% of all health care costs. The outcomes of critically ill patients are therefore important not only to the patients and their families but also to society. After ICU admission, the patient's outcome is completely based on the diagnosis and management of the primary illness and, in many cases, the presence or absence of multi-organ involvement [7, 8]. Electrolyte disorder pathophysiology, etiology, and clinical manifestation have been thoroughly reviewed [1]. Therefore, this study aimed to study the prevalence of electrolyte abnormalities among a group of hospitalized patients in Iraq.

## MATERIAL AND METHODS

### Study design and collection of data

This cross-sectional study involved patients admitted to ICU, RCU, and CCU in the Al-Sadar teaching hospital, Najaf, Iraq, between November 11, 2020, and April 2021. Patients without electrolyte data were excluded. The collected data included demographics, categories of ICUs admission, comorbidities, and laboratory results at admission. In addition, electrolyte data at ICU admission, during hospitalization, and other clinical data at admission were collected from an electronic medical database record.

### Statistical methods

The data were analyzed using Excel sheet version 16. Continuous variables are expressed as mean±SD, and categorical variables as numbers and percentages. The Chi-square test was used to analyze the categorical variables. Histograms were used to confirm the presence of dataset variable distribution. P-value<0.05 was considered statistically significant.

## RESULTS

The total number of patients was 200, with 145 males and 55 females. The male and female ratios were 3:1, 8:1 and 2.64:1 for ICU, CCU, and RCU, respectively. The mean ages of patients at ICU, CCU, and RCU were 60.7±18.96, 59.28±13.61, and 61.37±16.71 years, respectively. The averages of body mass index (BMI), fasting blood sugar (FBS), blood urea, and serum creatinine and the levels of serum sodium, potassium, ionized calcium, and chloride of the sample are summarized in Table 1.

**Table 1:** Demographic characteristics of the studied population.

Variables	Intensive care unit (ICU) n=52	Cardiac care unit (CCU) n=28	Respiratory care unit (RCU) n=120
Male	39	18	87
Female	13	10	33
Age (year)	60.7±18.96	59.28±13.61	61.37±16.71
BMI	21.09±1.68	22.78±2.15	22.83±3.16
FBS (mg/dl)	145.65±71.51	148.16±52.49	152.69±77.72
B.urea (mg/dl)	121.85±54.87	55.18±29.59	60.53±32.77
S. creatinine (mg/dl)	2.94±1.68	1.11±0.60	1.56±1.14
Na^+^ (mmol/l)	140.19±9.17	134.17±8.10	136.38±8.43
K^+^ (mmol/l)	3.18±0.62	2.72±0.86	3.43±0.60
Ionized Ca^2+^ (mmol/l)	0.94±0.34	0.69±0.23	0.99±0.21
Cl^-^ (mmol/l)	102.19±9.10	95.71±6.30	95.08±5.78

**Figure 1:**
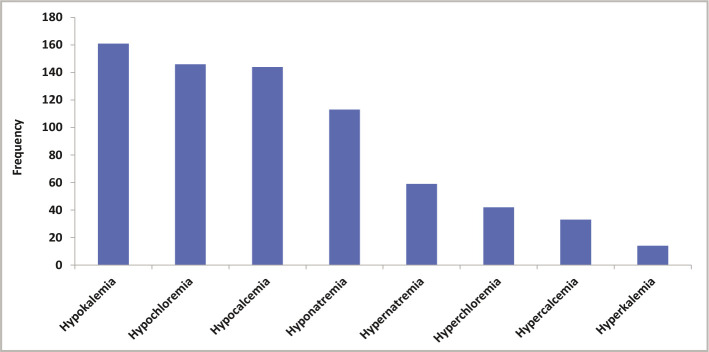
Percentage of electrolytes disorder in the studied population.

**Figure 2:**
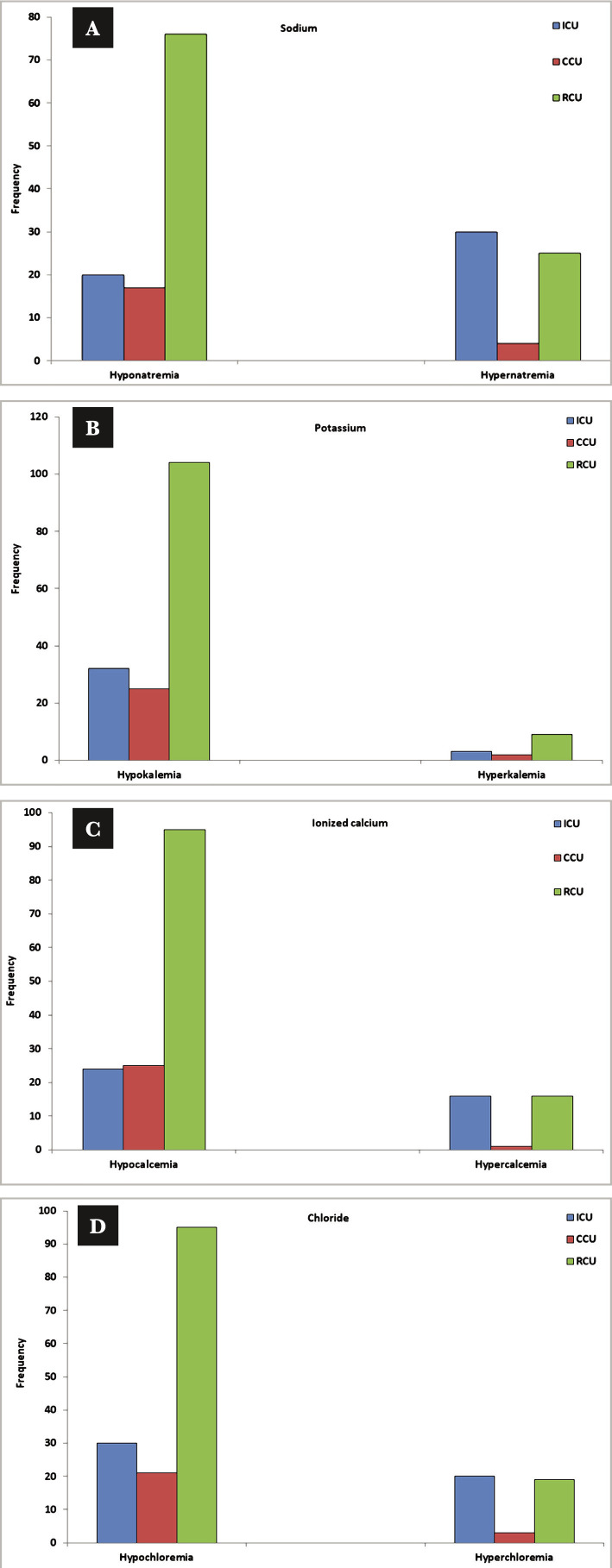
Prevalence of electrolyte disturbances during hospitalization in different categories of ICUs. A – sodium; B – potassium; C – ionized calcium and D – chloride.

**Figure 3:**
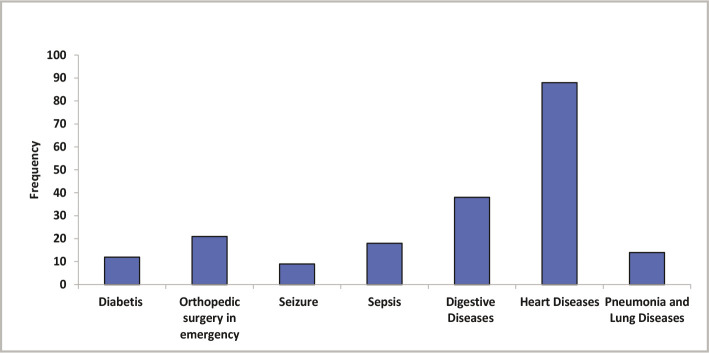
Co-morbidities among participants.

Table 2 shows the distribution of the type of hospitalization in the current study. The frequency of electrolyte abnormalities of sodium, potassium, ionized calcium, and chloride was highly significantly different between ICU, CCU, and RCU (P<0.001) (Table 2). The results indicated that hypokalemia was the most common disorder in hospitalized patients, as illustrated in Figure 1. The impact of hospitalization at ICU and during hospitalization at CCU and RCU on the serum electrolyte levels of patients is shown in Figures 2 A–D. The co-morbidities among the 200 study subjects are listed in Figure 3.

**Table 2:** The correlation between electrolytes and causes of hospitalization.

Variable/Status	Intensive care unit (ICU) n (%)	Cardiac care unit (CCU) n (%)	Respiratory care unit (RCU) n (%)	X^2^ (Chi-square)	P-value
Sodium (Na^+^) Normal Hyponatremia Hypernatremia Total	2 (3.85) 20 (38.46) 30 (57.69) 52 (26)	7 (25) 17 (60.71) 4 (14.29) 28 (14)	19 (15.34) 76 (63.33) 25 (20.83) 120 (60)	29.87	0.00000
Potassium (K^+^) Normal Hypokalemia Hyperkalemia	17 (32.69) 32 (61.54) 3 (5.769)	1 (3.57) 25 (89.28) 2 (7.14)	7 (5.8)3 104 (86.67) 9 (7.5)	26.32	0.0000
Ionized Ca^2+^ Normal Hypocalcemia Hypercalcemia	12 (23.07) 24 (46.15) 16 (30.76)	2 (7.14) 25 (89.28) 1 (3.57)	9 (7.5) 95 (79.17) 16 (13.34)	25.01	0.0000
Chloride (Cl^-^) Normal Hypochloremia Hyperchloremia	2 (3.84) 30 (57.69) 20 (38.46)	4 (14.28) 21 (75) 3 (10.71)	6 (5) 95 (79.17) 19 (15.84)	16.60	0.002

## DISCUSSION

The present study measured the frequency of electrolyte disturbances in hospitalized (ICU, RCU, and CCU) patients and related factors. This study enrolled a total of 200 patients, including 52 patients in ICU, 28 patients in CCU, and 120 in RCU in Al–Sadar teaching hospital in Najaf/Iraq. The prevalence, characteristics, and outcomes of electrolyte disorders were analyzed. Electrolyte abnormalities are common in ill patients. We measured the influence of electrolyte disturbances among critically ill patients.

Most importantly, a classification of hospitalized patients was established to evaluate the severity of electrolyte disorders and to predict hospital death. Wide varieties of electrolyte disorders occurred during hospitalization. A decrease or increase in the levels of electrolytes may be associated with an adverse prognosis. In addition, several diseases may cause different characteristics of electrolyte disturbances.

The current study found highly significant differences between ICU, CCU, and RCU groups regarding serum electrolytes such as sodium, potassium, calcium, and chloride levels (Table 2). Most of the disorders that occurred during hospitalization were observed in Figure 1. The analysis of RCU indicated that almost all participants in this group were suffering from pneumonia and lung diseases (Figure 3), including chronic obstructive pulmonary disease, pneumonia, and pulmonary hypertension. Within the group of RCU patients, combined electrolyte disorders were shown in 120 patients (60%). Hypokalemia was found in 104 (86.67%) patients, hypocalcemia in 95 (79%), hypochloremia in 95 (79%) patients, and hyponatremia in 76 (63%) patients. This agrees with another study [9] that measured the serum potassium and sodium in 64 patients with COPD and compared the results with 20 healthy volunteers. This study found a significant decrease in serum levels of K^+^ (3.39±0.96 mEq/L) and Na^+^ (133±6.86 mEq/L) in COPD patients when compared to the control groups (4.52±0.02 mEq/L, 142±2.28 mEq/L, respectively, P<0.05).

The present data on hypokalemia, hypocalcemia, hypochloremia and hyponatremia support other studies which found that patients with COPD are at a higher risk of decreased sodium, potassium chloride, and calcium levels [10]. The most likely explanations for the present data are using beta 2-adrenoceptor agonists and theophylline as the most common treatment in stable COPD. Unfortunately, these classes of treatments can induce electrolyte imbalance in respiratory issues and COPD patients [11]. Many factors might be responsible for producing electrolyte imbalances in COPD patients. These factors include malnutrition, diuretics use, syndrome of inappropriate antidiuretic hormone secretion (SIADH), renal insufficiency, activation of the renin-angiotensin-aldosterone system, and increased arginine vasopressin (AVP) [12]. A decreased potassium level leads to serious health problems, even death [13].

Furthermore, the current results are similar to another study [9], which reported respiratory acidosis with metabolic alkalosis caused by renal compensation as a common cause of hypochloremia in COPD patients. The researchers also suggested that those patients characterized by hypervolemia can impact electrolyte imbalance [14]. Consequently, these studies show that hypokalemia and other electrolyte imbalances may be common in severe COPD exacerbation patients, which should be adequately managed to avoid critical outcomes.

In CCU, many admitted patients are afflicted with heart diseases. The current study found that many patients at CCU had heart failure, unstable angina, myocardial infarction (MI), stable angina, ischemic stroke, and ST-segment elevation myocardial infarction (STEMI) (Figure 3). The frequency of hypokalemia (89%), hypocalcemia (89%), hypochloremia (75%), and hyponatremia (61%) in CCU are shown in Figure 3. The relationship between hypokalemia and myocardial infarction has been investigated in other studies [15]. Furthermore, many studies referred to the occurrence of cardiac arrhythmias in patients with acute myocardial infarction (AMI) and hypokalemia [16]. Thus, checking the potassium levels in a patient with heart problems is quite recommended, even if the potassium level was normal on admission [17].

Calcium ions play a vital role in the normal function of the cardiac muscle fibers [18]. Our results agree with several studies that found hypocalcemia impairs myocardial contractility, congestive heart failure caused by severe hypocalcemia [6], and cardiomyopathy in long-standing hypocalcemia. Additionally, coronary spasm resulting from hypocalcemia was described as the possible cause of chest pain in young patients initiating AMI [6, 19]. It was found that hypocalcemia is connected with the increased mortality of patients in the ICU [20]. Previous studies suggested the link between hypocalcemia and age, stating that hypocalcemic patients were adults and at higher risk of cardiovascular events, higher in-hospital mortality, and a lower rate of emergency revascularization compared with hypercalcemic patients. This is consistent with another study [21] that found high serum Ca^2+^ as a potential risk factor for a myocardial fraction [22]. In our study, 89% of cases had a low level of calcium compared to a very small percentage (4%) of patients with a higher level of calcium which might indicate a significant correlation between the risk of cardiovascular events and hypocalcemia.The current study measured ionized calcium instead of total calcium estimation to avoid calcium interference using gadolinium in the colorimetric assay of total calcium determination [23].

In CCU, hyponatremia can increase both morbidity and mortality in myocardial infarction patients. Also, myocardial infarction patients with low sodium levels have a low ejection fraction. For example, Pandey et al. found that 45% of patients with infarction had low levels of sodium, leading to an increased risk of death [24]. Moreover, the low sodium levels could be caused by impaired water excretion, leading to dilutional hyponatremia [25].

The level of hyponatremia (60%) in the current study agrees with another study, which found that hyponatremia was higher in non-survivors than survivors [24]. This study proposed that a low level of sodium in these patients suggests heart failure severity [26], but it could also be a heart failure outcome. Diuretic consumption is one cause of drugs producing hyponatremia [27]. Our results indicated that some CCU patients had higher levels of sodium.

In one study, the authors aimed to identify electrolyte prevalence and its association with mortality [28]. This study included 152 patients complaining of hypocalcemia, hyperkalemia, hyponatremia, hypernatremia, and hypercalcemia. Our study also found that 62% of ICU patients had hypokalemia, 58% had hypochloremia, 58% had hypernatremia, 46% had hypocalcemia, 39% had hyponatremia and hyperchloremia, 31% had hypercalcemia, and 6% had hypokalemia. However, our study found the prevalence of hypokalemia compared with other electrolytes. Sample differences, the difference between the subject's demographic descriptions and the purpose of patient's admission, may be the reasons for the differences between studies.

The prevalence of hypokalemia in the ICU is consistent with other studies that indicated a high percentage of hypokalemic patients in the ICU [29]. It was suggested that increased loss of K^+^ is the most likely cause of hypokalemia in patients suffering from diarrhea or taking diuretics [30]. For example, thiazide and loop diuretics increase sodium delivery, enhancing potassium secretion. Also, it can be caused by an increase in aldosterone secretion that is mediated by activating renin-angiotensin-aldosterone, associating volume depletion [30, 31].

Regarding hypochloremia, low chloride levels are related to diseases or interventions requiring treatment in the ICU [32]. For example, vomiting or renal loss are common causes of inducing hypochloremia. This supports our finding since almost all ICU admitted patients were suffering from gastrointestinal tract problems.

Our results on the prevalence of hypernatremia in the ICU are supported by other studies that observed higher sodium levels in ICU patients. Several factors might be associated with such circumstances, including decreased water ingestion, water loss, or excessive intake of sodium bicarbonate for metabolic acidosis correction [33].

## CONCLUSIONS

From this study, it can be assumed that combined electrolyte imbalance is abundant among hospitalized patients. This means the levels of electrolytes should be regularly assessed in hospitalized patients. Hypokalemia was evident in many RCU, ICU and CCU patients. It can lead to ventricular arrhythmias and increased mortality. Hypochloremia, hypocalcemia and hyponatremia were fairly common findings among hospitalized patients. The clinicians are advised to closely monitor these electrolyte changes and correct them as they seem to have negative effects on disease outcome and prognosis.

## Personal thanks

The authors would like to thank the department of statistics of Al-Sadar teaching hospital for their help during data collection.
